# Light People: Prof. Sir John Pendry, father of metamaterials, spoke about the future of meta

**DOI:** 10.1038/s41377-023-01082-w

**Published:** 2023-02-20

**Authors:** Chenzi Guo, Yu Luo

**Affiliations:** 1 Changchun Institute of Optics, Fine Mechanics and Physics, Chinese Academy of Sciences, Changchun, China; 2 School of Electrical and Electronic Engineering, Nanyang Technological University, Singapore, Singapore

**Keywords:** Metamaterials, Transformation optics

## Abstract

When consulting with the Marconi company in 1995, Prof. Sir John Pendry uncovered exotic structures that gave negative permittivity and negative permeability, respectively. In 1999, Prof. Pendry introduced split ring resonators (SRRs), and later in 2000, Prof. David Smith and Prof. Sheldon Schultz experimentally showed that periodic array of SRRs and continuous wires previously proposed by Prof. Pendry could exhibit simultaneously negative values of effective permeability and permittivity at the same frequency. Shortly after, Prof. Pendry revealed that a slab of material with simultaneous negative permittivity and permeability could challenge the Abbé diffraction limit on traditional lenses and focus all Fourier components of a point object onto a perfect image, leading to a “perfect lens”. The vision of a perfect lens attracted extensive research interest and opened a new field which was later widely known as metamaterials. Now two decades on, the explosion of metamaterials has revolutionized numerous researches in physics, materials science, chemistry, and engineering. To shed light on the research direction of metamaterials, *Light: Science & Applications* invited Sir John Pendry, father and living legend of metamaterials, to speak about the future of metamaterials. The original interview can be accessed in Supplementary video.


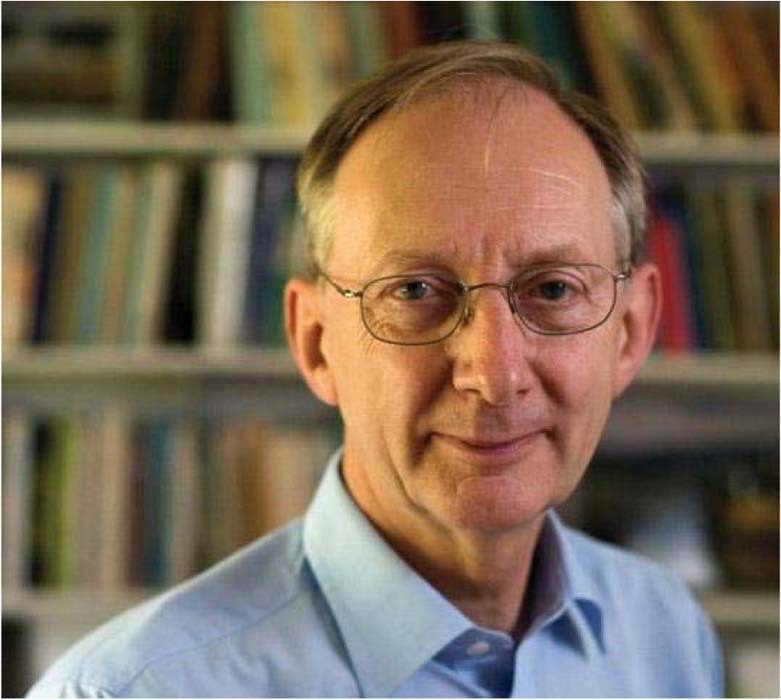


Professor Sir John Pendry is a condensed matter theorist at Imperial College, London. He received his Ph.D. from the University of Cambridge in 1969 and worked at Bell Labs from 1972–1973. He has held his professorship in the Blackett Laboratory of Imperial College, London, since 1981. Shortly after, he became the head of the Physics Department and Dean of Faculty of Natural Sciences. He is currently the Chair in Theoretical Solid State Physics. Prof Pendry is a Fellow of the Royal Society, the National Academy of Sciences of United States, American Academy of Arts and Sciences, the Institute of Physics (IOP), the Optical Society of America (Optica), American Physical Society (APS), etc. In 2004, he was knighted in the British Honours for his services to science.

Professor Pendry is one of the most highly cited British Scientists. He is recognized worldwide for his pioneering work on the structure of surfaces and their interaction with electrons and photons. He has also worked extensively on transport in disordered systems, where he produced a complete theory of the statistics of transport in one-dimensional systems. He founded the field of “metamaterials”, a concept for engineered structures whose electromagnetic properties depend on their internal structure rather than their chemical constitution. He discovered that a perfect lens manufactured from negatively refracting material would circumvent Abbeʼs diffraction limit to spatial resolution, which has stood for more than a century. His most recent innovation of transformation optics gives the metamaterial specifications required to rearrange electromagnetic field configurations at will. In its simplest form, the theory shows how we can direct field lines around a given obstacle and thus provide a cloak of invisibility. Several realizations of this concept have been built some operating at radar and others at visible wavelengths.

Professor Pendry has won numerous awards, including the Dirac Medal in 1996, the Royal Medal in 2006, the UNESCO-Niels Bohr gold medal in 2009, the Isaac Newton Medal in 2013, the Kavli Prize in Nanoscience in 2014, the Dan David Prize in 2016, etc. He holds honorary doctorates from five Universities in United States, France, Germany and Hong Kong.

**Q1: Your remarkable idea of realizing negative permeability showcased a practical way to make a negative refractive index lens, how did you come up with this radical idea?**

A1: Veselago had already said that a negative index material could do some focusing, but it was assumed that the focusing would be conventional and limited by the wavelength. I was intrigued by this lens because it doesn’t look like any other lens we’ve ever seen. It’s flat, and if you got the parameters right, impedance matched to vacuum, the geometrical aberration is zero. Chromatic aberration is another thing of course, it’s massively chromatic aberrating. But in the ray approximation, it seems to be perfect, it transmits everything perfectly, and it focuses everything, every ray perfectly in the Abbé sense. I then asked the question, ‘for all the rays that an ordinary lens can focus, this lens is perfect so what happens when the Abbé limit kicks in? Or in a more technical term, can this lens also focus evanescent waves which are permanently lost in all ordinary lenses?’. Luckily, I enjoy looking at the analytic structure of things, which tells you a lot about the bones of a problem. So I said, well, let’s look at the performance as a function of the k-parallel wave vectors. You will see all light rays that can be focused by a conventional lens are limited to k-parallel smaller than k_0_. Then, I kept asking myself if this new flat lens can break this limit, therefore being perfect for light with k-parallel beyond k_0_ as well. There was an anomaly: you would expect it to do something analytically, and what I expected was that it would do something very interesting analytically, but that it would stop focusing for some strange reason. So I looked at this, and to my surprise, the answer was: it doesn’t stop focusing. It just adds more and more Fourier components to give a ‘perfect’ image. I didn’t believe it. I thought, if I publish this, I’m going to be branded an idiot and my career will be finished in the optics. As you know, some people would have liked to see my career finished, so controversial was the result! But it wasn’t so. Eventually, we won through and persuaded people that this lens could do perfect focusing. I think there’s a moral in that. The moral is: if you see a slight disturbance in what you expect, don’t ignore it. Look at the disturbance, and ask why. Why is it so? Many other people must have looked at this lens and been busy, or for whatever reasons, ignored the disturbance, but I had some time that day, and I looked at it carefully.

**Q2: After you proposed the idea that negative refraction can make a perfect lens, many people didn’t believe you until the first experiment was done, but you continue to push this whole field. How did you cope with this pressure?**

A2: There was indeed a lot of pressure, more pressure than I’d ever had in my long career. I had done other things, which I think were quite interesting, but none had produced this sort of pressure. So it wasn’t something I was used to. What’s more, I had a rather big administrative job at that time. I was head of the department and if you’re doing that job, you have other people’s careers in your hands, so that comes first. I was hobbled, as we say, in that I couldn’t spend the time replying to my critics as I might have done in other circumstances. But eventually, I learned that controversy is a good thing for scientists. These people were arguing with me not because they were stupid, just that we were all grappling with a very interesting and complex problem. I think the controversy showed that the problem was interesting and drew a lot of people’s attention to it. So in the end, the controversy was good, particularly for me, because it was resolved in my favor. The only thing I regret was that some of the arguments were not very nice, and sometimes got personal. An important lesson for me was that, even though the other person might say some bad things, never do that yourself. If you see two people fighting in the street who you don’t know, you move on thinking they are equally bad, just as most people pass by scientific controversies where the fighting is personal and impolite: they will assume that both people are equally obnoxious, because they are fighting in the street! So never get into a fight in the street. I think it’s one moral, don’t answer bad language with more bad language. Just stick to the facts, if you’re wrong, admit it and if you’re right, just press on.

**Q3: We are very happy and lucky that you stuck to that and went through the controversy. So at the beginning of metamaterials, I guess you must have had a picture in mind about how the landscape of metamaterials will evolve in the next 20 years. Now looking back, any of your expected findings fail to be realized? And on the contrary, any findings that you didn’t expect at the very beginning?**

A3: Yes. I didn’t expect that the simple structures we began to work on at Marconi more than 20 years ago would prove so interesting. I am sometimes asked to predict the future and foolishly, sometimes I try to do so, but I will almost always be wrong. The surprising thing about metamaterials is that such a simple idea has been embedded in all sorts of modern technologies. Now, as the younger generation has taken over engineering, it’s in 5G, 6G, whatever. So I am very gratified and surprised. Although I had the first ideas in this subject, other people have contributed so much. It is as if I lit a flame: if you make a spark, have an idea, and that takes fire in other people, then that’s going to be very important. There have been some disappointments. For example, I had a project called “Better than Silver”. If we could find materials which are better than silver, there’s a lot more that could be done in the field, but they don’t seem to be forthcoming. So it’s a disappointment that we couldn’t do much better than silver. Actually doing as well as silver is something that is worth pursuing, because many metamaterial structures using silver don’t prepare the silver carefully enough. So silver is better than we think, but not as good as we would like it to be.

**Q4: So you mentioned the “Better than Silver” project, some would say that 2D materials could be engineered to reduce the loss. Do you think the graphene or other 2D materials can get chances to outperform silver?**

A4: Graphene is a remarkable material. I wouldn’t say it’s a competitor to metamaterials: they work on parallel tracks. They were invented at almost the same time and they each had a good ride, as you might say. The optical properties of graphene are quite remarkable, and I think there are a lot of opportunities there for plasmonics. But my view at this stage is that plasmonics is probably a field where the experiments should lead at this moment, because we must discover new materials, and that’s very difficult to do theoretically. Even if you do discover a theoretical material, you’ve then got to make it perfectly. And that’s the problem with silver. Very often it is prepared imperfectly with rough surfaces and so on, which destroys some of its properties. So yes, there’s a lot of potential in graphene, and many interesting things to do there. Of course, a lot of very clever people are working with graphene so we might see something there.

**Q5: Initially, metamaterials were first experimentally realized at microwave frequency. Then it was extended to the optical regime and UV range. So what do you think is the ultimate wavelength limit for metamaterials?**

A5: There are two obstacles there. One is that, when going to higher frequencies, the wavelength becomes shorter, and the engineering becomes more difficult. People now have very good control over engineering structures at the surface, so most of the metamaterial development in the optical region has been with 2D materials. Capasso and his ultra-thin lenses, for example, and very interesting they are too. But I think it will stay at the surface for a while, until people learn (as I’m sure they will) how to build up a surface into a 3D structure. There’s a lot that can be done with 2D metamaterials. So to summarize, the first challenge is manufacture. The second challenge is materials available to you. As you move to shorter wavelengths, the photons have more potential for exciting electrons, which means these structures are lossy. If you’re talking about negative refractive index, loss is a death sentence. So I don’t hold much hope for negative refractive index at extreme UV frequencies. But there are useful things you can do with lossy metamaterials, you will have to engineer structures that can tolerate that loss. It won’t be negative refraction but it might be some form of cloaking or controlling radiation.

**Q6: One of the initial motivations of metamaterials is to go beyond the diffraction limit. And it’s still a highly sought-after goal. So what do you think will be the ultimate diffraction limit in the metamaterial-based platforms?**

A6: Yes well, people are achieving very high focusing properties. The main application of these ideas has been to the problem of concentrating light: you can now concentrate light to about a nanometer. I rather suspect that will be a limit, because losses, manufacturing issues, and so on are kicking in at that point but focusing into a nanometer is still extremely useful. The first hidden hint that was taking place was in the work on the giant Raman resonance by Martin Fleischmann. Years after Fleischman’s discovery it turned out that the giant resonance was caused by focusing of light into the nanoscale gaps between rough regions on his silver surface. As I said in my IEEE paper, metamaterials and nonlinear effects are now becoming fashionable. People are now using this concentration of radiation to enhance nonlinear effects, to switch light on a very, very short time scale. So I think the enhancement of nonlinearity and enabling nonlinearity to take place without the necessity for very intense lasers, simply by concentrating a modest amount of power into a very small volume, that will enable very rapid switching. In fact, some of my colleagues here at Imperial, Riccardo Sapienza, and his group, are looking at switching. They can switch THz radiation on a time scale of one cycle, which is pretty fast.

**Q7: In early days, you predicted that on the extremely small scale, heat transfer will be significantly modified. And experimentally, lots of researchers are still throwing efforts into that. What are the challenges and chances?**

A7: There are several aspects of my work on heat transfer. Some of my early works showed that you could quantize heat transfer, and the thermal conductivity was quantized in units proportional to ℏ. That has been realized by a group in Caltech. What they did was to look in very narrow silicon nitride wires and measure their thermal conductivity. As they lowered the temperature, the number of vibrational modes available in the wire went down to one, then they saw a plateau in the thermal conductivity. So it’s very beautiful, but nobody’s repeated that experiment to my knowledge. But the other point is that using near field to actually enhance cooling of things, has been thought of being useful in, for example, gravitational wave detectors which require very intense laser beams. Light has to bounce around millions of times to get the resolution they need from the Fabry-Pérot cavity. So the mirror gets hot, and you have to cool it, but you cannot touch it otherwise you will make it wobble and induce noise in the signal. In such cases, you have to rely on radiative cooling, which is very poor. People have been thinking, can we use the near field to extract a little bit more from the mirror to cool it better? I think that’s very difficult, because the problem is, if you approach something close to the mirrors in the Fabry-Pérot cavity, not only do you enhance coupling of electromagnetic radiation through the near field, but also you enhance the van der Waals forces. At the same time, as you’re doing good things for electromagnetics, you are letting some low-frequency sound, i.e. vibrations, into the mirror as well as letting some heat out. It’s a trade-off, and I don’t think that has been resolved. But in situations where you are not too worried about making the thing vibrate, the near field is a possibility for cooling. The issue in the past has been how do you verify this theory experimentally? In the past the problem was to measure the temperature difference between two objects which are much less than a micron apart. Now there are techniques in use to measure the temperature difference between to objects close together, and with a very high accuracy, as well as spatial resolution. So these experiments are possible, and I’m looking forward to that being a developing field, though it’s been very slow in development.

**Q8: Through time, metamaterials have jumped out of the original four quadrants of permittivity and permeability to give the negative permittivity and negative permeability. And now it has been extended to explore loss and gain, also time-varying systems. Are there any possible future dimensions which can be added to interplay with metamaterials?**

A8: I talked about dimensions in the sense of space and time, and there are only four of those. However, Yu and I had a paper in which we generate artificial dimensions. So yes, you can do that. But before that happens, I think that we’re only just beginning to explore the time dimension, and I think that’s going to be very important. It’s very difficult to do it experimentally, because if you’re going to make something change with time, then to make it change very rapidly is a challenge. But I remember, when Eli Yablonovitch wrote his very famous paper on the photonic crystal, I wrote a piece for ‘News & Views’ in Nature. It was said that nobody would be able to make such a structure small enough to work for light. The engineering was just beyond the capability at that time, but now photonic crystals are fairly routine on an optical scale. I think the same about the time dependence of metamaterials. There are several things that time dependence can do for you. One, it will arouse tremendous interest in topological materials, with protected surface states, for example, which travel only one way and can’t be scattered backwards, because time reversal invariance is broken. But to really break time reversal invariance, you need to have a structure that is dependent on time. When we achieve that, topological ideas will bear much more fruit than they have done so far. Many topological systems rely on the absence of spin flip which is never perfectly absent. So that’s one thing that it will open up a whole area that theorists have been working on for a long time, but it is proving experimentally challenging. The other thing time-dependent systems do is that quantum mechanics becomes much more important than in static systems. If a system is static, when the photon enters, it may get absorbed, but it comes out with the same frequency if it comes out at all. On the other hand with a time-dependent system, you can make the photon hop about between frequencies. I am currently working with a colleague at the University of Exeter, Simon Horsley, on the quantum aspects of this. One of the things we find is that in some of these time-dependent structures, such as a moving grating, you can find analogies with Stephen Hawking’s radiation from a black hole. Years ago Hawking said that in a black hole the gravitational metric, which looks a bit like the refractive index, has a singularity. This is perhaps the only circumstance in which quantum mechanics meets general relativity. Hawking showed that the singularity will produce black body radiation with a temperature which we call the Hawking temperature. I remember when I was a graduate student, attending graduate lectures. The lectures were given by Paul Dirac one of the inventors of quantum mechanics, and at the back of the class in his wheelchair was Stephen Hawking. He was learning the quantum mechanics that he would use to make that prediction. That was something I remember well: two great men of science in the same room. So the quantum mechanical side is very interesting. Simon and I are proving quite a lot of theorems about when photons are conserved, when they’re not conserved, how energy is generated, and so on. I’ve talked about things moving and changing on a fantastical short time scale, the period of radiation. Obviously, you can’t make a physical object move at the speed of light, and you certainly can’t make it move faster than the speed of light. However, there’s another way of changing things, that is to keep the material static, but locally modulate it in a way phased from point to point, so the modulation moves in time. It’s like the phase velocity which isn’t limited by the speed of light. Now the modulations can move with any velocity from zero to infinity, because nothing is physically moving. It now becomes an experimental possibility that you might be able to make these structures. It may come to nothing, but I’m very optimistic. I’m very enthusiastic about the physics involved in these time-dependent structures and it occupies most of my time at this moment.

**Q9: Following that, I remember years ago, you mentioned about the dynamic Casimir force which is when the boundary moves very fast. It can convert a virtual photon from the vacuum fluctuation to a real photon. So to me, I think Hawking radiation is related to the dynamic Casimir force, wherein the moving boundary can generate the photon. Do you think there is any relationship between Hawking radiation and the dynamic Casimir effect?**

A9: Well, there’s a minor industry working on Hawking analogs. Hawking radiation occurs in extremely massive bodies and because the body is extremely massive the photons produced by the singularity have to climb through an intense gravitational field. By the time they leave the black hole, they are so depleted of energy, that it’s believed that no one will ever observe true Hawking radiation, because it’s just too weak. So the effort is focused on black hole analogs. Unruh has a model where you have a river flowing, and the bed of the river gets more and more shallow, so the velocity of the water increases with the shallowness. Then you have a point of no return, where something swimming in the water can never get back from crossing that singularity where the velocity of the water is so high. Even so, building these laboratory analogs is a challenge but there are people claiming to see Hawking-like radiation. One of the things that metamaterials can do in these time-dependent structures, if they could be realized, is spontaneously radiate and that may be one possibility for observing Hawking radiation, or pseudo-Hawking radiation, not true Hawking radiation.

**Q10: Metamaterials have been extended from their initial start in electromagnetics into their acoustic, mechanical, and thermal counterparts, all with fascinating demonstrations and applications. But I’m wondering what could be the physical border of metamaterials?**

A10: I think the concept applies to anything that obeys something like a second order differential equation. So we have acoustics, and even earthquake waves. One of my former postdocs, Sebastien Guenneau, has proposed a cloaking structure from earthquakes. That’s just amazing. So it will be foolish for me to say metamaterials should stop here, because if you’d asked me 20 years ago ‘are people going to think about metamaterials and earthquakes?’ then I’d say, ridiculous. But now, people are actually doing experiments on surface waves in the earth, controlling them with structures engineered in the ground. The sky is limit.

**Q11: What do you think are the most challenging problem for metamaterial, in the next three to five years? Or what do you think are the biggest problems of current metamaterial research?**

A11: That’s a question I ask myself. As a theorist, my main interest is looking at the time dimension. It’s an area with much difficulty experimentally. Relevant papers can date back to early 1950s, in which people looked at time-dependent structures, mainly electrical engineers who were thinking about microwave structures. But I think that so many technological advances happened recently that there’s a real possibility for experimental realization now. If we don’t see an experimental realization, all this theoretical work will sit on a shelf somewhere, and won’t do the useful things that other metamaterials have done. But I’m optimistic, and I think the opportunities opened by the time dimension are very great. Also, I think this time dimension will be assisted by the fact that metamaterials exist, not just in the electromagnetic domain (where the time dimension is difficult), but also in the acoustic domain (where the time scales are much longer). In the acoustic domain, you do have a chance to change materials on the time scale of a period. A while ago, I was in a conference in Venice, addressing some acoustic experts and trying to persuade them to do some of these experiments. A few of them said, yes, they will try. So we’ll see.

**Q12: Speaking of future applications of metamaterials, we all know that Facebook this year has changed its name to Meta, with the hope of creating a meta-universe. Though the meta over there are quite different from our metamaterials. But I’m curious about your opinion regarding this meta universe?**

A12: The only thing metamaterials have in common with the meta-universe is the word meta, which is merely a Greek word for saying beyond. What do I know about business? But I think the idea is silly, and it will come to nothing, and they will lose all their money.

**Q13: Some students will have a hard time when doing PhD, especially at their last year. Do you have any suggestions to share with young people before they pursue a PhD?**

A13: Let me first begin my answer by saying what they should NOT do. I was at a conference where several Nobel Prize winners were on the stage and answered this same question, and the answers were crazy. They said, what you should do is to ignore all the advice you receive, work on your own things, have your own ideas, and pursue them no matter what. And I thought, if the average young person in the audience did that, they would ruin their career. So here’s my advice. First of all, anybody who hopes to be a scientist or pursue a profession where skills are involved, should learn those skills very, very well. That takes about ten years. It’s similar when you want to play the piano or try painting: it takes about ten years to do it well. So the first thing is learning skills. Second, you’ve got to be passionate about what you’re doing. Otherwise, you won’t have the strength to stay and acquire those skills, because it’s a hard work. I would recommend young people to work on something that they really want to do. Third, far from ignoring what other people say, you should be very aware of what people around you are thinking and doing. If possible, you should find an advisor, a mentor, who will give good advice to you and perhaps stop you doing some silly things. I was lucky in my early years that I had some uncles, aunts and friends of the family who were interested in science, and they helped me a lot and encouraged my interest in science. So, talk to other people. Be aware of what they’re doing. Be aware of how “what you do” might help other people. Because if your work doesn’t key into what the whole world is doing, then you’re going to be ignored. Most people don’t like being ignored, nor do I. It’s not a rewarding situation for yourself. So to get that reward, you have to make your passions relevant to what other people were doing. That was a lesson I learned at Bell labs where many people there worked on very fundamental things, but at the same time, the labs created the tremendous technologies that we see in the world today.

**Q14: Some PhD candidates might encounter the situation that the advisor is asking them to do something, but they don’t find it passionate or rewarding enough. So in such situation, what’s your suggestion?**

A14: Haha, that’s almost a political question, isn’t it? Well, we all hope that our thesis advisor will be a person who responds to robust discussion. If as a student, you find that you’re being asked to do something you think is not right, then ask why and have a discussion. That will be part of your education. Most times, you’ll find you’re wrong because you’re an inexperienced student, and your supervisor has years of experience. But sometimes the supervisor will be wrong, and then you both learn something. So that’s the way rational discussion should take place, very different from fools arguing. It’s not my side or your side: it’s our side.

**Q15: Many famous scientists, like Einstein and Plank, play some music instruments. I know that you also play some instruments in your leisure time. Do you think music plays any role in your academic career?**

A15: Only indirectly. Music helps me to be more than a one-dimensional person. Through music, I meet other people, and have conversations about things that are quite different from science, and that’s important. As a whole person, science should not be the whole part of your life. On the other hand, music makes me realize one’s success in science is quite one-dimensional. Because if you turned your skills, as I do, to trying to play the piano, you might realize that in another theater of activity, quite a lot people are better than you. And it brings me to notice that most people don’t have the privilege of tremendous success in one area. Then what motivates these people? Why would you do something if you can’t be the best in the world? As G.K. Chesterton said, if a thing is worth doing, it is worth doing badly. Is there an intrinsic worth in something? I recalled a lovely story about Einstein. He was a keen but not terribly good violinist. When Einstein lived in Princeton, New Jersey, he once played music together with the great pianist Arthur Rubinstein who also lived in that region. After about 5 bars, Rubinstein stopped playing the piano and said, what’s the matter, Einstein? Can’t you count? There’s another story from a lady who heard Einstein play in a quartet in Berlin just before World War I, and she said it was such bad playing that all the four people in the quartet finished the music at different times. Yes, so music makes me realize: if a thing is worth doing, it’s worth doing badly.

**Q16: You and your wife are known as a fairy tale couple, and she has been supporting you all of your life. What do you think is the strongest support your wife ever gave to you? And if you don’t mind sharing, when is the moment that you decided to share your life with your wife?**

A16: I think the strongest support my wife gives is that she realizes I’m just another ordinary person. I think that’s important, because one needs to be able to look at success and failure in equal terms and face them both in a strong way. She enables me to do that. If I think that I have written a brilliant paper or something, she’s not overwhelmed. Equally, if I have a setback, she doesn’t think the worse of me for that. So it’s that strong relationship where you value each other for what you are, not for what you do. That’s the support which she gives to me. You asked when I decided to share life with her. Well, it’s not my decision, it’s her decision of course. When I decided to suggest that we get married, we had a holiday together in the Yorkshire countryside and I thought that was a good occasion to pop the question, as we had been dating for a couple of years or so. I had in mind a very beautiful spot in a valley where there is a ruined abbey near a river, which I knew from long ago. So I chose this spot, and I asked her, if she would marry me? I said, don’t answer the question yet. Go and talk to your mother and father, see whether they think it’s a good idea. And they did. So we got married.

## Supplementary information

Supplementary Information

